# Restoring natural killer cell activity in lung injury with 1,25-hydroxy vitamin D_3_: a promising therapeutic approach

**DOI:** 10.3389/fimmu.2024.1466802

**Published:** 2025-01-07

**Authors:** Johnny Amer, Ahmad Salhab, Mohammad Abuawad

**Affiliations:** ^1^ Department of Allied Sciences, Faculty of Medicine and Health Sciences, An-Najah National University, Nablus, Palestine; ^2^ Department of Biomedical Sciences, Faculty of Medicine and Health Sciences, An-Najah National University, Nablus, Palestine

**Keywords:** lung injury, 1, 25(OH)2D, NK cells, VDR, PD-1, PDL-1

## Abstract

**Background and aim:**

NK cells and NK-cell-derived cytokines were shown to regulate neutrophil activation in acute lung injury (ALI). However, the extent to which ALI regulates lung tissue-resident NK (trNK) activity and their molecular phenotypic alterations are not well defined. We aimed to assess the impact of 1,25-hydroxy-vitamin-D3 [1,125(OH)_2_D] on ALI clinical outcome in a mouse model and effects on lung trNK cell activations.

**Methods:**

Oleic acid (OA)-induced ALI in C57BL/6J mice and 1,25(OH)_2_D treatment 2×/2 weeks were performed. Lung tissue was harvested to assess alveolar I/II cell apoptosis and lung injury marker of Surfactant-Protein-D (SP-D). Pulmonary edema markers of epithelial sodium channel, cystic fibrosis transmembrane conductance regulator, and aquaporin 5 were assessed by RT-PCR. Lung trNK cells were assessed for activation markers of CD107a and NKp46, vitamin D receptor (VDR), and programmed cell death protein-1 (PD-1) via flow cytometry. The bronchoalveolar lavage fluid (BALF) obtained was investigated for soluble receptor for advanced glycation end products (sRAGE), inflammatory cytokines, soluble 1,25(OH)_2_D, and PDL-1. Naïve mice treated with DMSO (vehicle) were used as a control.

**Results:**

Flow cytometry analysis displayed a high apoptotic rate in alveolar I/II cells of threefold in ALI mice as compared to naïve mice. These findings were accompanied by elevated markers of pulmonary edema as well as lung injury markers of SP-D. Isolated lung trNK cells of the ALI mice exhibited reduced CD107a and NKp46 markers and cytotoxicity potentials and were correlated through significantly 2.1-fold higher levels of PD-1 and diminished VDR expressions as compared to naïve mice. BALF samples of ALI mice displayed high soluble PDL-1 and reduced soluble 1,25(OH)_2_D levels compared to naïve mice. 1,25(OH)_2_D treatment alongside OA led to a significant fourfold increase in the CD107a and NKp46 expressions to levels higher than the mice treated with the vehicle. Furthermore, 1,25(OH)_2_D ameliorates free radical scavengers of GSH, GPX, CAT, and GPx-1; decreased pro-inflammatory cytokines and soluble PDL-1; and increased soluble 1,25(OH)_2_D with amelioration in pulmonary edema markers and alveolar I/II apoptosis.

**Conclusion:**

Our results indicate 1,25(OH)_2_D’s potential therapeutic effect in preventing clinical outcomes associated with ALI via regulating NK cells through inhibiting inflammatory cytokines and alleviating levels of PDL-1 and 1,25(OH)_2_D released by lung tissue.

## Introduction

Acute lung injury (ALI) is a condition characterized by sudden and severe respiratory compromise, often progressing to acute respiratory distress syndrome (ARDS) (1). It represents a critical form of pulmonary dysfunction with significant morbidity and mortality rates (2). The pathophysiology of ALI involves a complex interplay of inflammatory mediators, oxidative stress, and endothelial dysfunction (3). Alveolar epithelial cell damage and the infiltration and activation of inflammatory cells such as neutrophils and macrophages contribute to releasing pro-inflammatory cytokines and chemokines (4). This inflammatory cascade leads to increased vascular permeability, pulmonary edema, and impaired surfactant function, culminating in severe hypoxemia and respiratory failure (5).

1,25(OH)_2_D, the active form of vitamin D_3_, plays a crucial role beyond its classical functions in calcium homeostasis and bone metabolism (6). It exerts pleiotropic effects on various tissues and cells throughout the body, including those in the respiratory system (7). In recent years, research has revealed its potential therapeutic implications in conditions such as ALI and ARDS (8). One of the key mechanisms by which 1,25(OH)_2_D exerts its effects is through binding to the vitamin D receptor (VDR), which is expressed in a wide range of cells, including epithelial cells, immune cells, and endothelial cells within the lung (9). Upon binding to VDR, 1,25(OH)_2_D modulates gene expression, leading to immunomodulatory, anti-inflammatory, and cytoprotective effects (10–13). While 1,25(OH)_2_D has garnered attention as a potential therapeutic agent in ALI (11–17), its specific effects on natural killer (NK) cells and the molecular pathways regulating these effects remain largely unknown. NK cells are innate immune cells that play a crucial role in host defense against pathogens and in immune surveillance against tumor cells (18). Emerging evidence suggests that 1,25(OH)_2_D may modulate NK cell function, potentially impacting the immune response in ALI (19). It is hypothesized that 1,25(OH)_2_D may exert its effects through various molecular pathways involved in immune regulation, including the modulation of cytokine production, cell signaling pathways, and gene expression profiles within NK cells. In this study, we aimed to assess the molecular effects of 1,25(OH)_2_D on ALI mouse models and their impacts on resident lung NK cells’ activity in an approach to attenuate inflammation and improve clinical outcomes in ALI.

## Methods

### Animal ethics and study design

The animals used in our experimental procedures were housed in a barrier facility and received care according to the An-Najah National University ethical regulations and NIH guidelines. The institutional animal care ethics committee of the An-Najah National University approved all animal protocols under the ethics number 2024-02-01. Mice groups: Group 1: Naive mice with no treatment of either OA and 1, 25(OH)_2_D treatment and received only intraperitoneal (i.p.) injection of DMSO (0.01%) as vehicle, twice weekly for 2 weeks. Group 2: Naive mice treated with 1,25(OH)_2_D (100 ng/kg), i.p. injection twice a week for 2 weeks. Group 3: ALI mice treated with OA (150 μL/kg), intravenous (i.v.) injection twice a week for 2 weeks. Group 4: ALI mice treated parallelly with both OA (150 μL/kg) and 1, 25(OH)_2_D (100 ng/kg). Each mouse group had *n* = 11.

### Acute lung injury animal model

The acute lung injury model was introduced in 12-week-old male wild-type C57BL/6J mice (weighing 25 ± 1.5 g) by i.v. injection of oleic acid (OA; Merck, O1008-1G >99% purity, as determined by LC/MS analyses) at a concentration of 150 μL/kg twice a week for 2 weeks using special microsyringes to ensure precise and accurate delivery of the small injection volume (3.5 μL/25 g mouse weight). DMSO and 1,25(OH)_2_D treatment was prepared fresh daily in phosphate-buffered saline (PBS) at a concentration volume of 100 μL.

### Animal experimental setting

Mice and diet were weighed, and water intake was measured daily. Prior to sacrifice, animals were anesthetized by 5% isoflurane inhalation for 10 s before cervical dislocation. On the day of sacrifice, lung bronchoalveolar lavage fluid (BALF) extracts were assessed for sRAGE, soluble PDL-1, soluble 1,25(OH)_2_D, and pro-inflammatory cytokine profile assessments by enzyme-linked immunosorbent assay (ELISA). As described below, mRNA was obtained from the lung for gene expression assessment. Lung tissue-resident NK (trNK) cells were assessed using flow cytometry.

### Bronchoalveolar lavage fluid extraction from lung tissues

The trachea was surgically exposed and intubated with a syringe catheter. The lungs went through lavage with 1 mL of pre-warmed PBS five times. A total of 5 mL of BALF was obtained from each mouse, and cells in BALF were pelleted by centrifugation (500 g for 10 min at 4°C). Cells were then resuspended in cold PBS, counted, and immediately applied to subsequent analyses. The supernatants were stored at −80°C for BAs measurements.

### Cytokine assessment in BALF

A multiplexed sandwich enzyme‐linked immunosorbent assay‐based technology (Cat# MHSTCMAG-70K; R&D Systems) was used to simultaneously determine the concentration of multiple cytokines of IL-1β, TNF-α, IL-6, and IL-4 in BALF. In addition, sRAGE concentrations were assessed using an ELISA kit (Cat# Abcam; ab197745), and soluble PDL-1 and 1,25(OH)2D concentrations were assessed using Cat# Abcam; ab210971 and Cat# Abcam; ab213966, respectively. All reagents and samples were brought to room temperature (18–25°C) before use. A volume of 100 µL of each standard and BALF sample was added into appropriate wells and incubated for 2.5 h at room temperature with gentle shaking. The solution was discarded, and wells were washed four times with 1× wash solution. Notably, washing was done by filling each well with wash buffer (300 µL) using a multi-channel Pipette or auto washer. After washing, the liquid was completely removed at each step, which is essential for good performance. A volume of 100 µL of 1× prepared detection antibody was added to each well for 1 hour at room temperature with gentle shaking. A volume of 100 µL of prepared streptavidin solution was added to each well for 45 min at room temperature with gentle shaking. A volume of 100 µL of TMB One-Step Substrate Reagent (Item H) was added to each well for 30 min at room temperature in the dark with gentle shaking. Finally, 50 µL of stop solution (Item I) was added to each well. Absorbance was read at 450 nm immediately using an ELISA reader (Tecan M100 Plate Reader). In addition, cells were counted using light microscopy in multiple fields, and the average number of cells per field was calculated.

### Lung tissue-resident NK cell isolations

Under deep ether anesthesia, mice were euthanized by isoflurane, USP 100% (INH), and then the lung was removed, and a part of it was transferred to a Petri dish that contains 5 mL of DMEM medium (Biological Industries; Cat# 01-055-1A). The tissues were thoroughly dissected by stainless steel mesh, the cells were harvested with the medium and added to 50-mL tubes containing 10 mL of DMEM, and then carefully cells were transferred to new tubes that contained Ficoll (Abcam; Cat# AB18115269). Tubes were centrifuged for 20 min at 1,600 rpm at 20°C. The buffy coat (interphase layer) in each tube was transferred to a new tube for another 10 min, at 1,600 rpm at 4°C. After the second centrifuge, the pellet in each tube was suspended in 1 mL of DMEM for the NK isolation kit (Stem Cells; Cat# 19665). Lung trNK cells were phenotyped as CD45^+^CD3^-^NK1.1^+^CD49a^+^CD69^+^CD103^+^ by flow cytometry, as mentioned below.

The viability of the lung trNK cells (10^6^/100 μL) was assessed by propidium iodide (PI) (A35110, R&D Systems) staining. PI-negative cells were considered viable when the mean viability rate of the cells was above 90%.

### Flow cytometry

All used antibodies were incubated with the isolated cell suspensions (1:100) at 4°C for 45 min; cells were washed with 2× PBS with 1% FCS before the secondary antibody (1:100) at 4°C for 45 min if required. Primary mouse antibodies used are anti-CD45 (ab10558, abcam), anti-CD3 (ab33429, abcam), anti-NK1.1 (ab289542, abcam), anti-CD49a (142605, BioLegend), anti-CD69 (104505, BioLegend), anti-CD103 (121405, BioLegend), anti-CD107a [lysosomal-associated membrane protein-1 (LAMP-1) (ab24170, abcam)], anti-NKp46 (ab83946, abcam), anti-PD-1 (ab214421, abcam), and anti-VDR (ab3508, abcam). Isotype IgG labeled with the relevant fluorochrome was used as a control for each antibody. Prior to flow cytometry analysis, cells with a count of 10^6^/100 μL were assessed for their viability measured by PI (A35110, R&D Systems). PI-negative cells were considered viable, and the mean viability rate of the cells was 92.7% ± 1.5%. All stained cells were examined on a flow cytometer (BD LSR Fortessa™, Becton Dickinson, Immunofluorimetry Systems) and analyzed by FCS Express 7 by *De Novo* Software for Flow Cytometry.

### TrNK cytotoxicity assay

K562 cells, a human leukemia cell line, were used as target cells in a cytotoxicity assay to evaluate the activity of lung trNK cells. The assay was performed by coculturing NK cells with K562 cells at a 4:1 effector-to-target ratio for 6 h as described by Lisovsky et al. (20) After the coculture incubation, the cells were stained with NK cell markers, including NK1.1, and Annexin V to assess apoptosis. The alveolar cells were identified as negative for NK1.1 expression, confirming their non-NK cell phenotype. Apoptosis was evaluated by detecting Annexin V binding.

### RNA isolation, cDNA preparation, and real-time PCR

A total cellular count of 10 μg/μL RNA (purity 98%) determined using a Nanodrop ND-1000 spectrophotometer (Nanodrop Technologies, Wilmington, DE) was isolated from all mouse groups (*n* = 11) using 2 mL of TRI reagent (Bio Lab; Cat# 90102331). The samples were centrifuged (1,400 rpm) for 15 min at 4°C to collect RNA supernatant. For RNA precipitation, the supernatant of each sample was transferred to a new microcentrifuge tube, and 0.5 mL of isopropanol (Bio Lab; Cat# 16260521) was added and incubated at 25°C for 10 min. The tubes were then centrifuged (12,000 rpm) for 10 min at 4°C, the supernatants were removed, and 1 mL of 75% ethanol was added to the pellets prior to centrifugation (7,500 rpm) for 5 min. The pellets were air-dried at room temperature for 15 min, 50 μL of DEPC was added, and the samples were heated for 10 min at 55°C. cDNA was prepared with a High-Capacity cDNA Isolation Kit (R&D; Cat# 1406197). Real-time PCR was performed with TaqMan Fast Advanced Master Mix (Cat# 4371130, Applied Biosystems) to quantify SP-D, ENaC, CFTR, and AQP-5 gene expressions with normalization to the expression of the housekeeping gene GAPDH. Cycling conditions for the Thermofisher one-step RT-PCR kit involved RT steps for 30 min at 50°C and denaturation for 15 min at 95°C. Furthermore, the reaction mixture was incubated for 40 cycles of 94°C for 30 s, 60°C for 30 s, and 72°C for 1 min, followed by 72°C for 10 min. Data analysis was carried out using the QuantStudio™ 5 Real-Time PCR System (Cat# A34322, Applied Biosystems).

### Lung oxidant and antioxidant activity assay

The homogenized lung tissue was centrifuged at 9,000 *g* for 15 min. The separated supernatant was used for oxidative stress assessment of malondialdehyde (MDA) and free radicals’ scavengers of glutathione (GSH), glutathione peroxidase (GPX), catalase (CAT), and glutathione peroxidase activity (GP×1) were determined using an ELISA kit according to the manufacturer’s instructions.

### Alveolar epithelial cell isolation and phenotyping

Both type I (AT-I) and type II (AT-II) alveolar epithelial cells were isolated from mice lungs, as described by Chen et al. (21), and sorted using FACSAria III Cell Sorter (BD FACSAria), as defined by Gonzalez et al. (22) Mice were euthanized, and the lungs were harvested and inflated with a collagenase-based digestion buffer to separate the alveolar epithelial cells. The lung tissue was minced and incubated in the digestion buffer at 37°C with constant agitation to dissociate the cells. After digestion, the tissue was filtered through a 70-µm mesh to remove debris. The resulting single-cell suspension was subjected to differential centrifugation to enrich epithelial cells. AT-I cells were distinguished by positivity for anti-mouse aquaporin 5 (Abcam; Cat# 315855) while AT-II cells were identified using anti-mouse epithelial cell adhesion molecule (EpCAM, Abcam; Cat# ab221552) and anti-mouse surfactant protein C antibody (SP-C, Abcam; Cat# 211326). The staining was confirmed by comparable staining patterns following the use of isotype IgG labeled with the relevant fluorochrome (isotype negative control). AT-1 and AT-II viability was assessed by PI staining according to the kit instructions (A35110, R&D Systems). Samples ≥90% PI-negative cells were considered viable. Apoptosis was evaluated with annexin V (A35110, R&D Systems) staining. Early apoptotic cells were defined as annexin V^+^PI^−^ cells, and late apoptotic cells were defined as annexin V^+^PI^+^ cells. All stained cells were examined by flow cytometry with a BD LSR Fortessa Cell Analyzer (Becton Dickinson, Immunofluorometry Systems) and analyzed using FCS Express 7 with *De Novo* Software for flow cytometry.

### Statistical analysis

Statistical differences were analyzed with a two-tailed unpaired Student’s *t*-test (for comparisons between two groups) or two-way analysis of variance (ANOVA) with GraphPad Prism 9.0 (GraphPad Software, La Jolla, CA). A *t*-test of *p*-value ≤0.05 is considered statistically significant and was calculated as the difference in means between two variables. Results are presented as mean ± SD or as average means of experimental replicates ± SD.

## Results

### 1,25(OH)_2_D alleviates lung injury markers outcome in a mouse model of ALI

**Figure 1:**
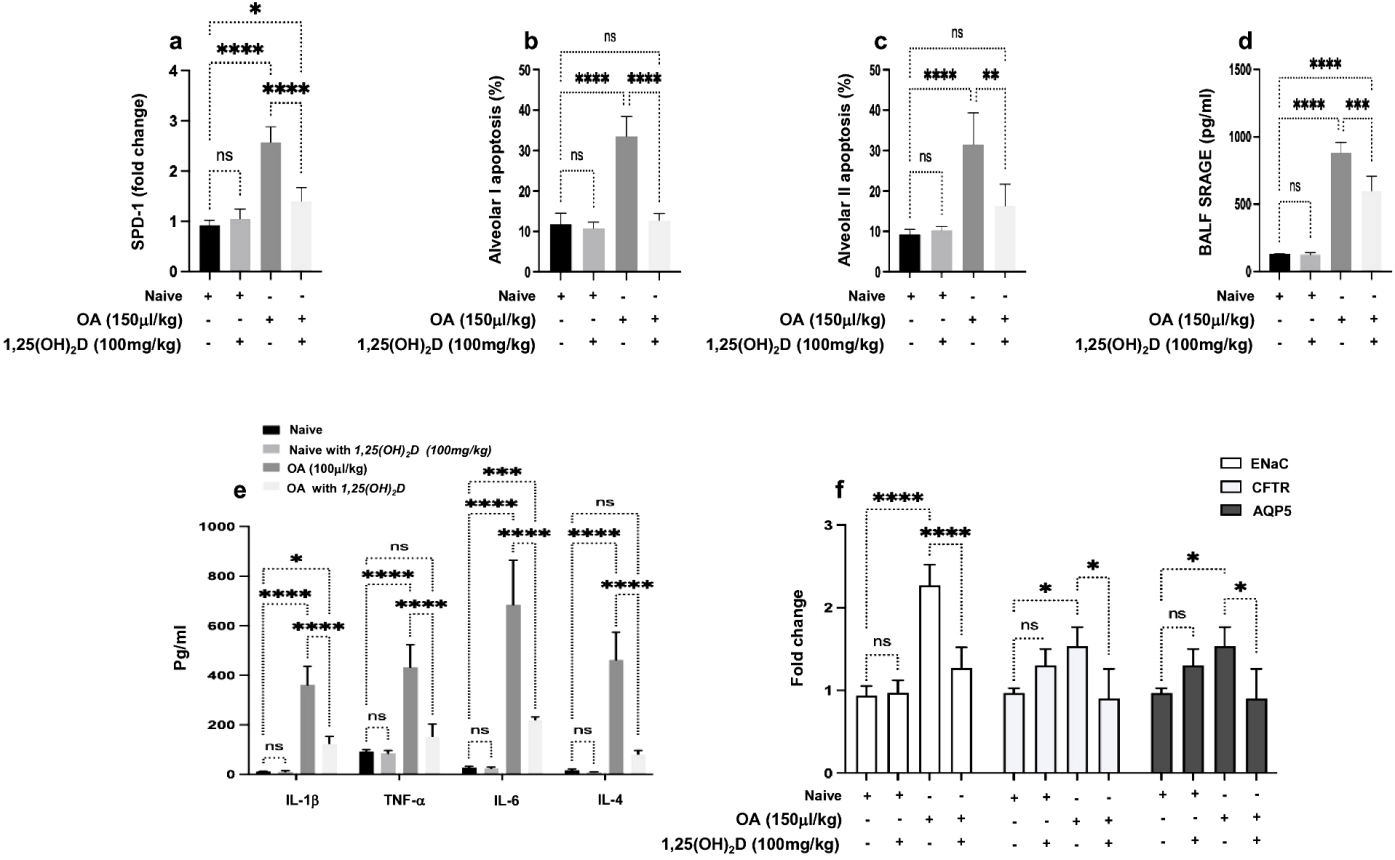
**(A)** Quantitation SP-D mRNA expression in lung tissues obtained from all mouse groups. The data are presented as fold change relative to the expression in naïve mice treated with the DMSO (0.01%), with GAPDH as the normalization housekeeping gene. Alveolar cells of **(B)** type 1 and **(C)** type 2 were assessed for percent apoptosis (annexin-V^+^/PI^−^). A multiplexed sandwich enzyme-linked immunosorbent assay-based technology was used to simultaneously determine the concentration of **(D)** sRAGE and **(E)** pro-inflammatory cytokines of IL-1β, TNFα, IL-6, and IL-4. **(F)** RT-PCR of ENaC, CFTR, and AQP5 alveolar fluid transport and data represented as fold change compared to naïve mice. Data are represented as averages ± SD (*n* = 11 per group). Significance was determined using Newman–Keuls two-way analysis of variance (ANOVA), *p < 0.01, **p < 0.001, ***p < 0.0001, ****p < 0.00001.

1,25(OH)_2_D effects on OA-induced ALI mice were assessed for their potential therapeutic effects via determining ALI markers of inflammatory, fibrotic, oxidative, and apoptotic markers. Surfactant Protein D (SP-D), a marker for interstitial lung diseases (ILDs) and ARDS (23), exhibited a significant increase of 2.5-fold in mice administered with 0.15 mL/kg OA. These elevations showed a decrease of 1.47-fold following treatment with 100 ng/kg 1,25(OH)_2_D (**Figure 1A**). No effects of 1,25(OH)_2_D were seen in the naive mice. Moreover, alveolar cell apoptosis, an important mechanism in ALI progressions, was assessed in isolated alveolar I (AT-1) and alveolar II (AT-II) cells. **Supplementary Figure S1** displays representative flow cytometry dot blot analysis of the lung cell isolate that was used in identifying AT-I and AT-II cells using gating strategies and specific antibodies as indicated in Materials and Methods. Flow cytometry analysis displayed a high apoptotic rate in both AT-1 and AT-II cells threefold as presented in **Figures 1B, C**, respectively (*p* < 0.0001). These findings were restored in the mice treated with 1,25(OH)_2_D to levels comparable to naïve mice treated with vehicle. Impaired and extensive epithelial injury results in the inflammatory phase of ALI (24). Therefore, to further characterize whether 1,25(OH)_2_D modulates the inflammatory outcome, sRAGE, involved in the inflammatory response during ALI effects, as well as pro-inflammatory cytokines, were assessed in BALF samples obtained from our mouse groups. **Figures 1D, E** display a significant increase in sRAGE, IL-1β, TNF-α, IL-6, and IL-4 in the OA-treated mice of 4- to 10-fold (*p* < 0.001). Nonetheless, 1,25(OH)_2_D treatments were shown to attenuate BALF sRAGE levels through a 1.46-fold reduction as compared to naïve mice. Moreover, 1,25(OH)_2_D decreased pro-inflammatory cytokines, suggesting the anti-inflammatory effects of 1,25(OH)_2_D in defending against insults of OA and reducing inflammatory markers associated with ALI.

Pulmonary edema results from a combination of both increased fluid filtration and impairment of transepithelial Na+ transport (25). Therefore, we sought to assess whether 1,25(OH)_2_D could inhibit OA-augmented edema formation. Epithelial sodium channel (ENaC), cystic fibrosis transmembrane conductance regulator (CFTR), and aquaporin 5 (AQP5) were assessed. **Figure 1F** illustrates an increase in ENaC, CFTR, and AQP5 levels in the OA-treated mice of 2.3-, 1.7-, and 1.67-fold, respectively (*p* < 0.01). In contrast, 1,25(OH)_2_D treatments reduced ENaC expressions by twofold, while CFTR and AQP5 showed an inhibition following the 1,25(OH)_2_D treatment to levels comparable to the naïve mice.

The effects of OA on mice and lung weight were assessed daily within the experiment’s duration. OA caused a reduction in weight relative to mice treated with vehicles. Conversely, mice receiving both OA and 1,25(OH)_2_D demonstrate a restored weight to levels comparable to naïve mice receiving the vehicle (**Supplementary Figure S2A**). Concerning lung weight, mice treated with OA significantly increased lung weight. On the other hand, 1,25(OH)_2_D treatment exhibits a substantial reduction in lung weight, comparable to naïve mice receiving the vehicle (**Supplementary Figure S2B**).

### Biomarker of oxidative stress and antioxidant defense

Recent reports regarding the canonical role of oxidative stress in the development of ALI (26). The imbalance between the production of free radicals and their elimination by antioxidants was described as the major protective mechanism against OA-induced-ALI (27). To test the antioxidant effects of 1,25(OH)_2_D, we initially examined MDA activity in serum and lung tissue samples following OA treatment. **Table 1** demonstrates an increase in serum as well as lung tissue MDA of 2.8- and 3.8-fold, respectively, as compared to naïve mice (*p* < 0.05). However, administration of 1,25(OH)_2_D along OA caused a significant reduction of MDA levels in serum and lung tissue of 1.8- and 5.3-fold, respectively (*p* < 0.05). To further define the association of the antioxidant effects of 1,25(OH)_2_D in preventing OA-induced ALI, the levels of free radical scavengers GSH, SOD, CAT, and GSH-Px were assessed in both serum and lung tissue samples. **Table 1** illustrates that these antioxidant markers were significantly lower in serum and lung tissue samples from OA-treated mice than in naïve mice (*p* < 0.05). However, when mice of ALI were treated with 1,25(OH)_2_D, a significant increase in the levels of 1.4- to 2.0-fold of these antioxidant markers was noticed compared to ALI mice with OA alone (*p* < 0.05). Overall, our findings suggest that 1,25(OH)_2_D boosts the antioxidant defense system against OA-induced ALI and emphasizes the role of free radicals in ALI pathogenesis.

**Table 1:** A multiplexed sandwich enzyme-linked immunosorbent assay-based technology was used to determine the concentration of MDA, GSH, SOD, CAT, and GSH-PX in serum and lung tissue.

Group		MDA (mmol/L) (nmol/g tissue)	GSH (mmol/mL) (nmol/g tissue)	SOD (U/L) (U/g)	CAT (U/L) (U/g)	GSH-Px (U/L) (U/g)
Naive	Serum Lung	4.28 ± 0.27 3.18 ± 0.22	157.23 ± 10 141. ± 4	241 ± 55 200 ± 18	60.45 ± 3.5 82.8 ± 6	50.32 ± 3.5 40.23 ± 6
Naive + 1,25(OH)_2_D	Serum Lung	5.22 ± 0.29* 4.52 ± 0.26	166 ± 9.3 132 ± 11.6	233.7 ± 25 210 ± 0.3	68.5 ± 3.6* 79.6 ± 7*	54.35 ± 1.5* 49.6 ± 3.3
OA	Serum Lung	12.19 ± 0.35* 12.3 ± 0.15*	92.3 ± 45* 81.3 ± 9*	154.5 ± 16* 60 ± 9*	22 ± 0.18* 33.3 ± 0.25*	12.2 ± 6* 21.5 ± 6.3*
OA + 1,25(OH)_2_D	Serum Lung	6.76 ± 0.6^#^ 2.31 ± 0.9^#^	133.3 ± 9^#^ 127.35 ± 13^#^	181.35 ± 8^#^ 167 ± 12^#^	71.3 ± 5.1^#^ 67 ± 2.3^#^	60.3 ± 1.3^#^ 36.25 ± 3.2^#^

Data are represented as averages ± SD (*n* = 11 per group). Significance was determined using *t*-test analysis, ^*^p-value <0.05 between the naïve group and OA group, #*p*-value <0.05 between the OA group and OA treated with 1,25(OH)_2_D group.

### Molecular pathways of 1,25(OH)_2_D improving lung NK cell cytotoxicity in ALI

**Figure 2:**
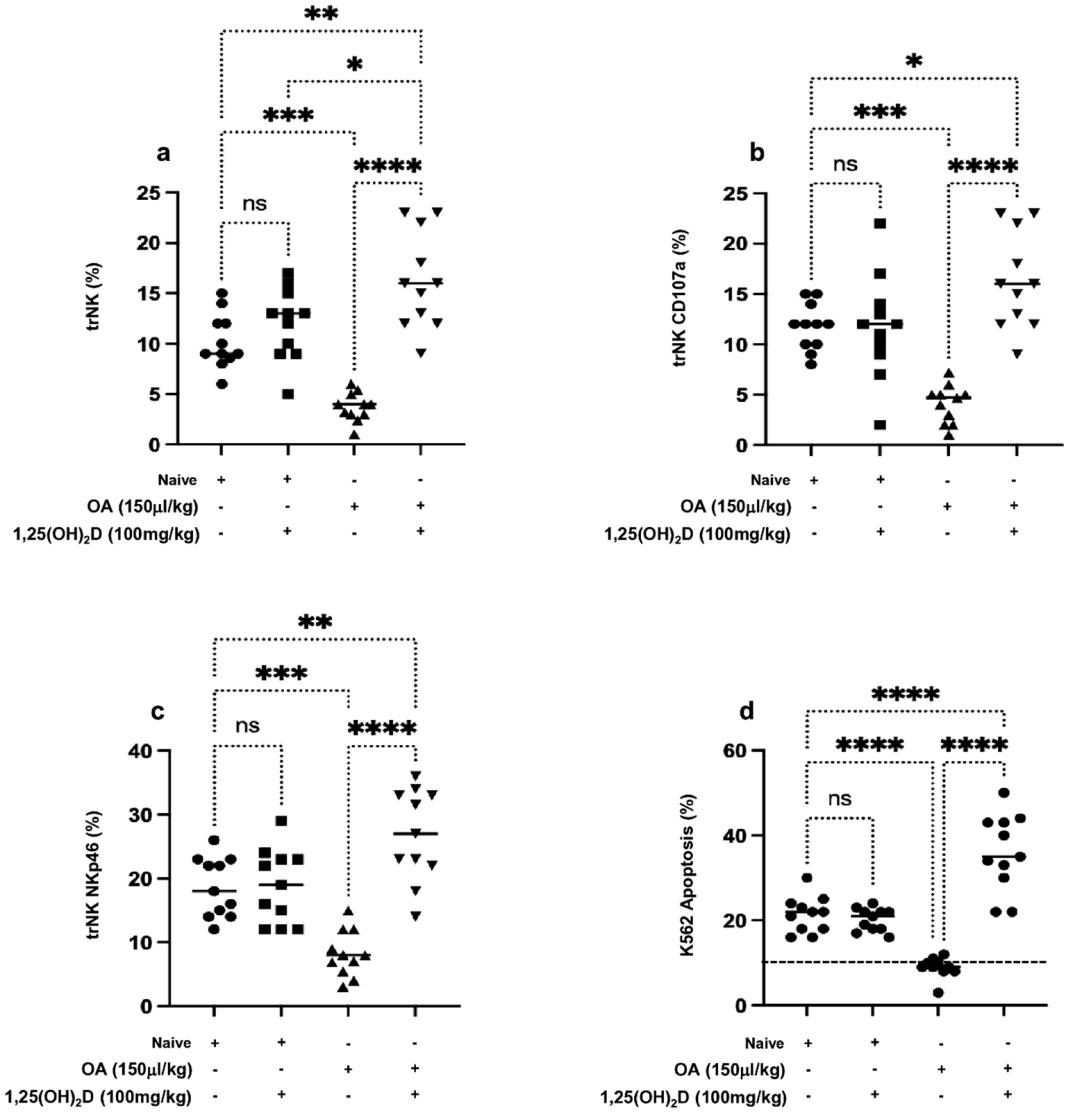
Flow cytometry analysis of **(A)** lung trNK cells and activation markers of **(B)** CD107a and **(C)** NKp46 assessed in all mouse groups. **(D)** Cytotoxic assay of lung trNK cells through cell incubation with K562 as a target cell in the ratio of (4:1) for 6 h, then K562 cell apoptosis was assessed through annexin-V^+^/PI^−^ detection by flow cytometry analysis. Data are represented as averages ± SD (*n* = 11 per group). Significance was determined using Newman–Keuls two-way analysis of variance (ANOVA), *p < 0.01, **p < 0.001, ***p < 0.0001, ****p < 0.00001.

Diffuse alveolar damage (DAD) is the dominant pathological feature of ALI (28). Moreover, one of the pathological features of ALI includes uncontrolled inflammatory response during immune and neutrophil movement (29), a process that is in part shown to be controlled by NK cells through producing pulmonary CXCL1 and CXCL2 (30); however, NK cell’s function and factors influencing its activity in ALI are still underestimated. We attempted to assess lung NK cells’ cytotoxic role in ALI progressions following OA treatment. **Supplementary Figure S3** displays illustration on flow cytometry phenotype analysis of lung trNK cell isolates and tested markers for NKp46 and CD107a. **Figure 2A** displays the distribution of lung trNK cells represented as counts and percentages assessed by flow cytometry. Lung trNK cells showed a severe reduction in their percentages by 6.0-fold following OA treatment as compared to the naïve mice. In contrast, following 1,25(OH)_2_D treatment, lung trNK cells showed a comparable and elevated distribution percentage as seen in the naïve mice treated vehicle.

To further investigate trNK cells’ activity in correlation to their elevated count distribution in lung tissue following 1,25(OH)_2_D, trNK cell activation markers of CD107a and NKp46 were assessed. Our findings reveal a marked decrease in the expression levels of CD107a and NKp46 markers in mice treated with OA of 2.5-folds (**Figure 2B**) and 2.1-folds (**Figure 2C**), respectively, compared to those treated with DMSO alone. Furthermore, 1,25(OH)_2_D treatment alongside OA led to a significant 4-fold increase in the CD107a and NKp46 expressions to levels higher than the mice treated with the vehicle. Furthermore, we associate trNK activity with their potential cytotoxicity effects through a coculture assay of these cells with K562 cells, and the K562 cell’s apoptotic rate was investigated. **Figure 2D** demonstrates that K562 cells cocultured with lung NK cells of ALI had an apoptosis percentage of 10% ± 2.2%, similar to K562 monoculture represented in the dotted horizontal line. Lung trNK cells of ALI treated with 1,25(OH)_2_D increased the K562 apoptosis rate to 38.4% ± 5.4%. Conversely, K562 cocultured with lung trNK cells from the naïve mice treated with 1,25(OH)_2_D remained with the same apoptotic rate as compared to naïve mice receiving the vehicle (*p* = ns). Our findings suggest 1,25(OH)2D’s effect on promoting trNK cell activation and its cytotoxic potentials, consequently inducing K562 killing. However, these results do not fully explain the factors affecting NK cell impairment in ALI.

To assess phenotypic alterations that occurred in lung NK cells of ALI, we assessed PD-1 expressions and VDR on NK cells. PD-1-positive NK cells have been shown to exhibit a weaker antitumor function than that of PD-1-negative NK cells in lung cancer (31). However, their role in regulating NK activity in ALI is still not addressed. Moreover, the VDR is shown to be expressed in most immune cells (32), while its expression on lung NK cells is not documented, and how vitamin D enhances immune effector pathways of NK cells is still unknown.

**Figure 3:**
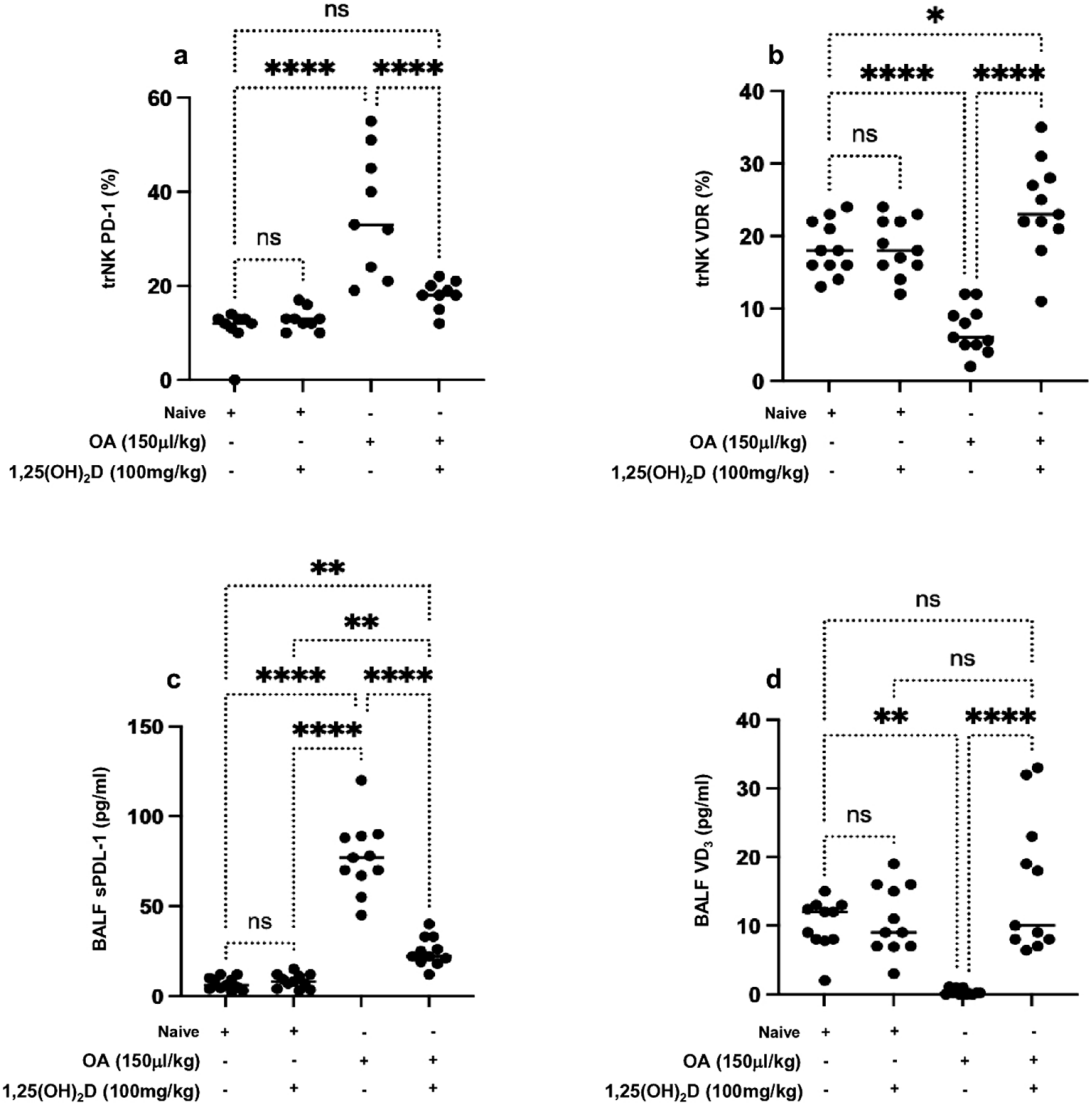
Flow cytometry analysis of lung trNK cells markers of **(A)** PD-1 and **(B)** VDR. A multiplexed sandwich enzyme-linked immunosorbent assay-based technology was used to determine the concentration of **(C)** soluble PDL-1 and **(D)** VD_3_ in BALF. Data are represented as averages ± SD (*n* = 11 per group). Significance was determined using Newman–Keuls two-way analysis of variance (ANOVA), *p < 0.01, **p < 0.001, ***p < 0.0001, ****p < 0.00001.

**Figure 3A** demonstrates that lung trNK cells of the ALI mouse model exhibited significantly 2.1-fold higher levels of PD-1 than naïve mice. Moreover, 1,25(OH)_2_D treatment displayed a significant decrease in lung trNK cell PD-1 expression levels to levels similar to naïve mice treated or untreated with 1,25(OH)_2_D. On the other hand, lung NK cells expressed diminished VRD in the ALI mouse model and had expressions of 8% ± 1.9% compared to 18% ± 4.1% in naïve mice (**Figure 3B**, *p* < 0.05). Lung trNK cells isolated from the ALI treated with 1,25(OH)_2_D exhibited VDR expressions of 26% ± 7.2%. Naïve mice with and without 1,25(OH)_2_D treatment had similar VDR on lung trNK cells. These results, in part, could indicate (1) 1,25(OH)_2_D as an immune effector in enhancing trNK cell activity, (2) alteration in lung phenotypic alteration in the ALI mouse model, and (3) inverse association of trNK cell activity with PD-1 and positive association between lung VRD and trNK activity. Nevertheless, our current data are still inadequate for explaining regulatory factors influencing lung trNK cell activity in ALI. Soluble factors present in the lung that can regulate trNK cell activity include TGF-β (33), prostaglandins produced by alveolar macrophages (34), and pulmonary surfactant (35).

Considering the alteration in lung NK cell expressions of PD-1 and VDR in ALI, we sought to determine levels of soluble PDL-1 in BALF samples as the major ligand of PD-1 that are shown in many studies to be dominantly expressed on alveolar cells and alveolar macrophages (36). Moreover, we suggested assessing for soluble 1,25(OH)_2_D, as suggested by Zheng et al., and its role in attenuating lung injury via stimulating epithelial repair, reducing epithelial cell apoptosis, and inhibiting induced epithelial-to-mesenchymal transition (37). **Figure 3C** indicates highly detected levels of soluble PDL-1 79.5% ± 7.6% pg/mL in the ALI compared to 9.7% ± 2.5% pg/mL in the naïve mice (*p* < 0.05). In contrast, reduced levels of soluble 1,25(OH)_2_D in BALF samples of ALI were noticed with a concentration of 1.4% ± 0.4% pg/mL as compared to 12.3% ± 3.0% (**Figure 3D**). The ALI mouse model treated with 1,25(OH)_2_D caused a reduction in soluble PDL-1 of 2.1-fold and an elevation in soluble 1,25(OH)_2_D of 10.3-fold to comparable levels seen in the naïve mice. Overall results indicate 1,25(OH)_2_D’s potential therapeutic effects in preventing clinical outcomes associated with ALI via regulating NK cells through inhibiting inflammatory cytokines and alleviating levels of PDL-1 and vitamin D released by lung tissue.

## Discussion

Inflammation in ALI can be triggered through both exogenous pathways, including toll-like receptors (TLRs), and endogenous pathways related to members of the damage-associated molecular pattern (DAMP) released by dead cells or local inflammatory cells (29). However, the involvement and association of 1,25(OH)_2_D, the active form of vitamin D_3_, in the regulatory role of NK cells in a mouse model of ALI was not well established prior to our current study. We investigated the role of 1,25(OH)_2_D in modulating the activity of lung trNK cells within the context of ALI induced by OA. ALI and its severe manifestation, ARDS, present intricate challenges in clinical management, necessitating a deeper understanding of the underlying pathophysiology and potential therapeutic interventions (38). Our study sheds light on the immunomodulatory effects of 1,25(OH)_2_D on NK cells and its implications for ALI management.

NK cells are pivotal components of the innate immune system, tasked with surveilling and eliminating virally infected cells and tumor cells (39). Upon infection, rapid NK cell infiltration into the lungs occurs, the amplitude of which is determined by the extent of inflammation and damage (40). Activated NK cells kill infected cells and produce pro-inflammatory cytokines and chemokines to recruit cells of the adaptive immune system (41). However, their dysregulation or impairment can exacerbate tissue injury and inflammation in various pathological conditions, including ALI (42). Our findings unveil the immunomodulatory effects of 1,25(OH)_2_D in alleviating trNK cell function via CD107a and NKp46 and phenotypic alteration through the assessment of crucial receptors, such as PD-1 and VDR on trNK cells that are important in defining the regulatory destination of lung trNK cells and consequently influencing the pathogenesis of ALI.

Recent evidence shows that immune cells and cytokines secreted by immune cells play an irreplaceable role in the pathogenesis of acute lung injury (29). Cytokines such as TNF-α and interleukins (mainly IL-1β and IL-6) are important mediators in the development of ARDS, contributing to augmented vascular permeability and organ dysfunction (43). Our data evidently showed the effects of 1,25(OH)_2_D in ameliorating the overall rate of alveolar fluid transport reflected by the tested expression of ENaC, NKA, and CFTR. Reactive oxygen and nitrogen species (RONS) can modify or damage ion channels, such as epithelial sodium channels, which alter fluid balance, a phenomenon that was suggested by our study to be reversed through the boosting of antioxidant markers of GSH, SOD, CAT, and GSH-Px following 1,25(OH)_2_D treatment. Mokrá et al. showed that the flow of protein edema fluid into the alveoli can lead to the inactivation of surfactants and the loss of the protective layer on the alveolar surface, thus destroying the surface cell structure (44).

Overall, our study unveils the multifactorial clinical outcome of ALI on regulating NK activity in particularly soluble mediators in BALF samples, while 1,25(OH)_2_D alleviates this outcome and attenuates NK cytotoxicity, suggesting its role in the resolution of inflammation mediated in part by NK cells.

Further translational and clinical studies are warranted to offer promising insights into 1,25(OH)_2_D-based therapies for ALI management.

## Conclusion

Our study findings indicate an association between 1,25(OH)_2_D treatment and reduced clinical outcomes associated with alveolar cell apoptosis, inflammation, and pulmonary edema in ALI. This improvement correlated with an increase in VDR and a decrease in PD-1 expression on trNK cells, potentially influencing NK cell activity. These results underscore the immunomodulatory effects of 1,25(OH)_2_D, linked to its anti-inflammatory and antioxidative properties. While these findings are promising, further research is necessary to determine whether these associations directly contribute to improved outcomes and to fully understand the therapeutic potential of 1,25(OH)_2_D in ALI.

## Data Availability

The datasets presented in this study can be found in online repositories. The names of the repository/repositories and accession number(s) can be found in the article/**Supplementary Material**.

## Supplementary material

Supplementary Figure 1 Flow cytometry dot blot analysis of **(A)** mice lung cells isolate gated as “gate 1” including viable cells as illustrated by SSC-A and FSC-A. **(B)** Dot plots of cells obtained from gate 1 representing AT-1 as indicated as positive staining for anti-mouse aquaporin 5 (AQP5) and gated as “gate 2”. (C) Dot blot analysis illustrating double positive cells obtained from gate 2 for Annexin-V (ANNV) and Propidium Iodide (PI) as indicators for early apoptosis (ANNV+PI-) and late apoptosis (ANNV+PI+) as indicate in materials and methods. Flow cytometry dot blot analysis of **(D)** mice lung cells isolate gated as “gate 1” including viable cells as illustrated by SSC-A and FSC-A. **(E)** Dot plots of cells obtained from gate 1 representing AT-II as a double positive staining for anti-mouse epithelial adhesion molecule (EpCAM) and anti-mouse surfactant protein C antibody (SP-C) and gated as “gate 2”. **(F)** Dot blot analysis illustrating double positive cells obtained from gate 2 for Annexin-V (ANNV) and Propidium Iodide (PI) as indicator for early apoptosis (ANNV+PI-) and late apoptosis (ANNV+PI+) as indicate in materials and methods.

Supplementary Figure 2
**(A)** At the termination of the experiment, mice were sacrificed, and their weight was measured in grams. **(B)** Following the experimental timeline, lungs from each mouse were carefully harvested, and their weights in grams were measured. Data are represented as averages ± SD (*n* = 11 per group). Significance was determined using Newman–Keuls two-way analysis of variance (ANOVA), ***p < 0.001, ***p < 0.0001, ****p < 0.00001*.

Supplementary Figure 3 Representative flow cytometry dot blot analysis of **(A)** NK cells following isolation by NK isolation Kit following Buffy coat isolates following Ficoll-Paque, cells were gated as “gate 1”. **(B)** Cells obtained from gate 1 were stained for NK1.1-FITC (NK maker) and CD3-APC (pan T-cell marker) for assessing NK purification and a “gate 2” was set on these cells. These cells were also positive for CD45 (data not shown). TrNK cells from gate 2 were identified for CD103+CD69+ cells, a gate was set on these cells as “gate 3”. **(C-E)** NK cells from gate 3 were identified for NKp46 and CD107a.
