# Supplementary figures and images for: Restoring natural killer cell activity in lung injury with 1,25-hydroxy vitamin D3: a promising therapeutic approach
Source: Front Immunol. 2025 Jan 7;15:1466802. doi: 10.3389/fimmu.2024.1466802 (PMC11746039; doi:10.3389/fimmu.2024.1466802)

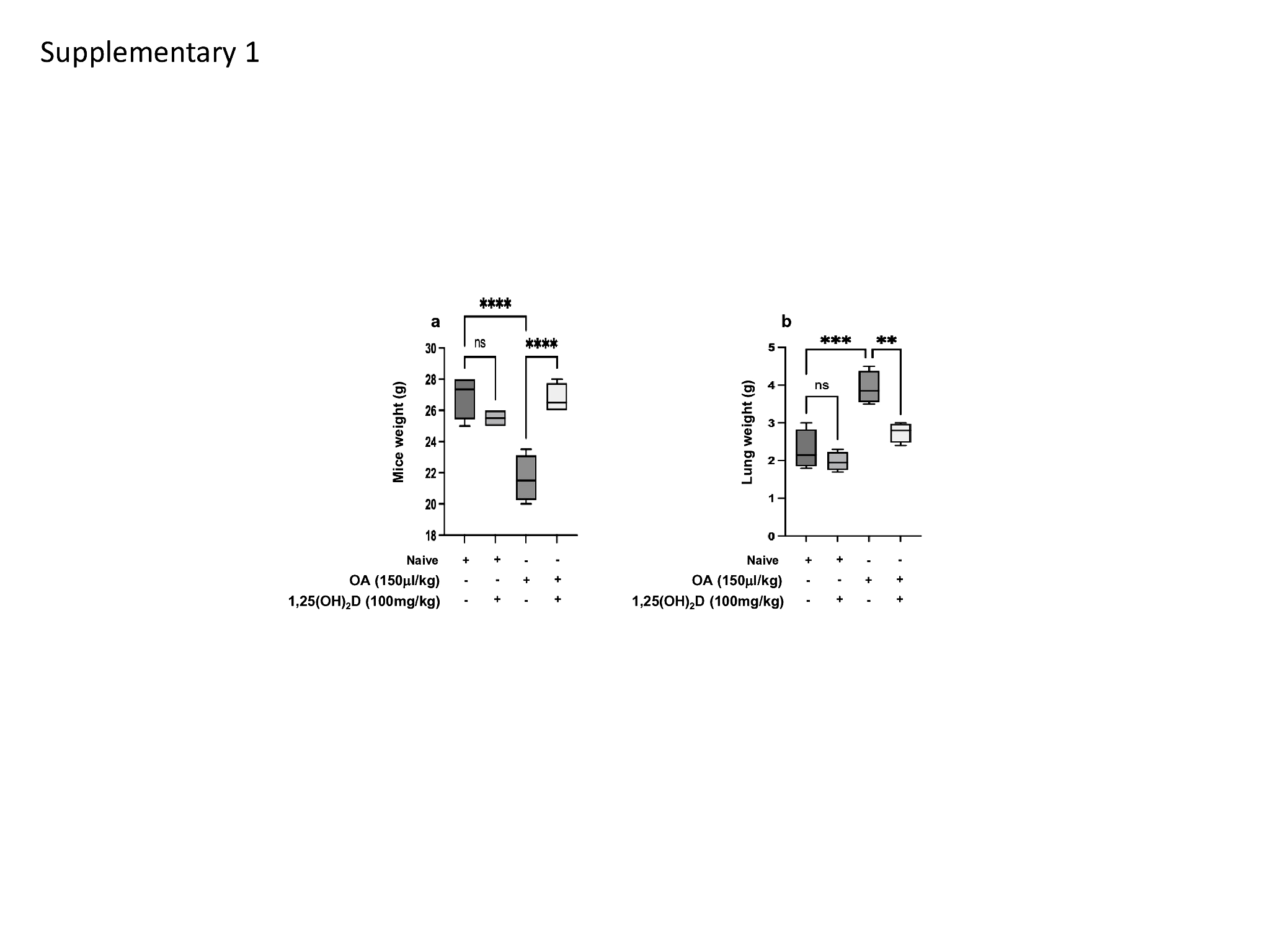

Supplement: Supplementary Figure 1 — Flow cytometry dot blot analysis of (A) mice lung cells isolate gated as “gate 1” including viable cells as illustrated by SSC-A and FSC-A. (B) Dot plots of cells obtained from gate 1 representing AT-1 as indicated as positive staining for anti-mouse aquaporin 5 (AQP5) and gated as “gate 2”. (C) Dot blot analysis illustrating double positive cells obtained from gate 2 for Annexin-V (ANNV) and Propidium Iodide (PI) as indicators for early apoptosis (ANNV+PI-) and late apoptosis (ANNV+PI+) as indicate in materials and methods. Flow cytometry dot blot analysis of (D) mice lung cells isolate gated as “gate 1” including viable cells as illustrated by SSC-A and FSC-A. (E) Dot plots of cells obtained from gate 1 representing AT-II as a double positive staining for anti-mouse epithelial adhesion molecule (EpCAM) and anti-mouse surfactant protein C antibody (SP-C) and gated as “gate 2”. (F) Dot blot analysis illustrating double positive cells obtained from gate 2 for Annexin-V (ANNV) and Propidium Iodide (PI) as indicator for early apoptosis (ANNV+PI-) and late apoptosis (ANNV+PI+) as indicate in materials and methods. [file Image1.jpg]

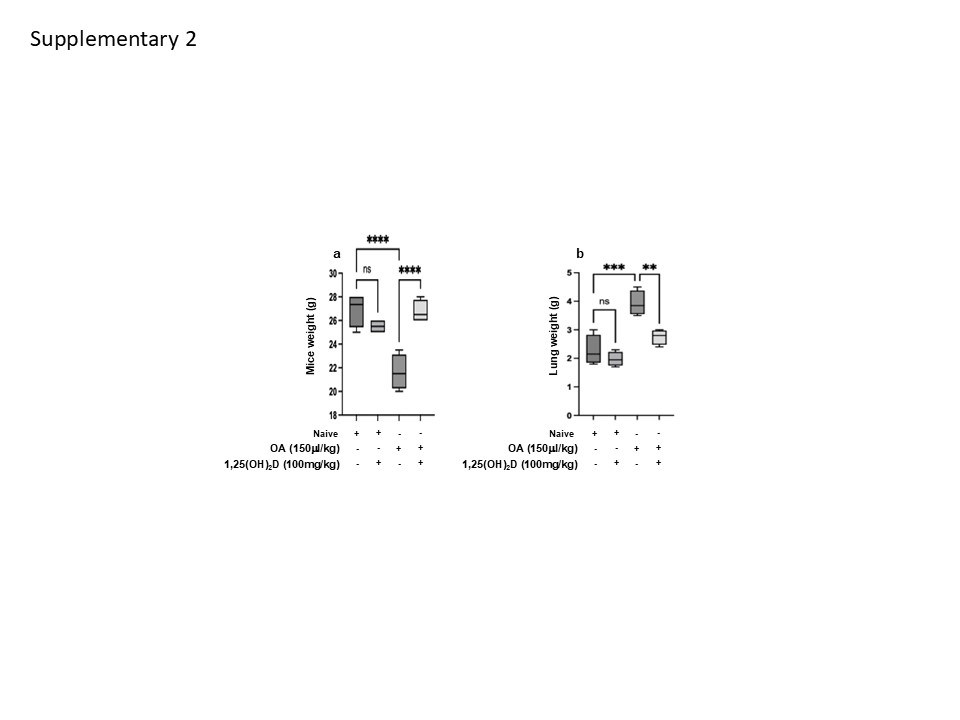

Supplement: Supplementary Figure 2 — (A) At the termination of the experiment, mice were sacrificed, and their weight was measured in grams. (B) Following the experimental timeline, lungs from each mouse were carefully harvested, and their weights in grams were measured. Data are represented as averages ± SD (n = 11 per group). Significance was determined using Newman–Keuls two-way analysis of variance (ANOVA), **p < 0.001, ***p < 0.0001, ****p < 0.00001. [file Image2.jpeg]

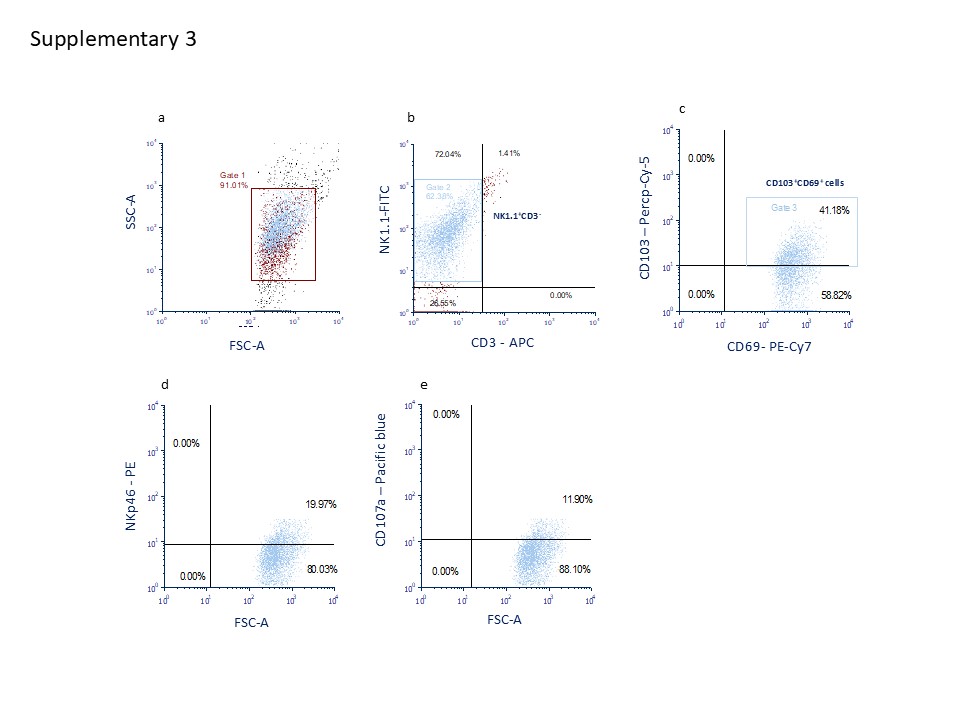

Supplement: Supplementary Figure 3 — Representative flow cytometry dot blot analysis of (A) NK cells following isolation by NK isolation Kit following Buffy coat isolates following Ficoll-Paque, cells were gated as “gate 1”. (B) Cells obtained from gate 1 were stained for NK1.1-FITC (NK maker) and CD3-APC (pan T-cell marker) for assessing NK purification and a “gate 2” was set on these cells. These cells were also positive for CD45 (data not shown). TrNK cells from gate 2 were identified for CD103+CD69+ cells, a gate was set on these cells as “gate 3”. (C-E) NK cells from gate 3 were identified for NKp46 and CD107a. [file Image3.jpeg]
